# Integrated mRNA and miRNA transcriptome analysis reveals a regulatory network for tuber expansion in Chinese yam (*Dioscorea opposita*)

**DOI:** 10.1186/s12864-020-6492-5

**Published:** 2020-02-03

**Authors:** Yunyi Zhou, Shuzhen Luo, Saba Hameed, Dong Xiao, Jie Zhan, Aiqin Wang, Longfei He

**Affiliations:** 1 College of Agriculture, Guangxi University, Nanning, 530004 People’s Republic of China; 2 Guangxi Key Laboratory for Agro-Environment and Agro-Product Safety, Nanning, 530004 People’s Republic of China; 3 Guangxi Colleges and Universities Key Laboratory of Crop Cultivation and Tillage, Nanning, 530004 People’s Republic of China

**Keywords:** Yam tuber, *Dioscorea opposita*, Expansion, mRNA, Small RNA

## Abstract

**Background:**

Yam tuber is a storage organ, derived from the modified stem. Tuber expansion is a complex process, and depends on the expressions of genes that can be influenced by environmental and endogenous factors. However, little is known about the regulatory mechanism of tuber expansion. In order to identify the genes and miRNAs involved in tuber expansion, we examined the mRNAs and small RNAs in *Dioscorea opposita* (Chinese yam) cv. Guihuai 16 tuber during its initiation and expansion stages.

**Results:**

A total of 14,238 differentially expressed genes in yam tuber at its expansion stage were identified by using RNA sequencing technology. Among them, 5723 genes were up-regulated, and 8515 genes were down-regulated. Functional analysis revealed the coordination of tuber plant involved in processes of cell events, metabolism, biosynthesis, and signal transduction pathways at transcriptional level, suggesting that these differentially expressed genes are somehow involved in response to tuber expansion, including CDPK, CaM, CDL, SAUR, DELLA, SuSy, and expansin. In addition, 541 transcription factor genes showed differential expression during the expansion stage at transcriptional level. MADS, bHLH, and GRAS were involved in cell differentiation, division, and expansion, which may relate to tuber expansion. Noteworthy, data analysis revealed that 22 known tuber miRNAs belong to 10 miRNA families, and 50 novel miRNAs were identified. The integrated analysis of miRNA-mRNA showed that 4 known miRNAs and 11 genes formed 14 miRNA-target mRNA pairs were co-expressed in expansion stage. miRNA160, miRNA396, miRNA535 and miRNA5021 may be involved in complex network to regulate cell division and differentiation in yam during its expansion stage.

**Conclusion:**

The mRNA and miRNA datasets presented here identified a subset of candidate genes and miRNAs that are putatively associated with tuber expansion in yam, a hypothetical model of genetic regulatory network associated with tuber expansion in yam was put forward, which may provide a foundation for molecular regulatory mechanism researching on tuber expansion in *Dioscorea* species.

## Background

Yams (*Dioscorea spposita*) are monocotyledonous plants belonging to the family *Dioscoreaceae*, and tuber is its harvested organ. Tuber originates from the expansion of underground stem, is suitable for nutrients storage, with many large parenchyma cells. Tuber morphogenesis and starch, along with accumulated proteins are two main processes of tuber growth [1]. The tuber morphogenesis of yam can be divided into three stages: initiation stage, expansion stage, and maturation stage. The expansion stage can be divided into three periods: early expansion stage, middle expansion stage, late expansion stage [2, 3]. Tuber morphogenesis is a complex physiological process regulated by heredity, environment, and hormones [4]. Great efforts have been made to explore the physiological factors affecting the morphogenesis of yam tubers. The short-day treatment tended to promote tuber growth at the primary tuber growth stage of the plant, and bulbil development at the rapid tuber growth, but the responses varied among species and cultivar [5, 6]. Endogenous hormones including gibberellins (GA), acetic acid (IAA) and abscisic acid (ABA) performed a key role at the beginning of tuber expansion stage, and trans-zeatin (tZ), jasmonic acid (JA) were also involved in tuber expansion [2, 7, 8]. Exogenous hormones have been used to study the mechanism of tuber expansion, GAs could promote tuber expansion and yield through in vitro and in vivo treatment [9, 10]. Exogenous GA application combined with ABA has promoted microtuber growth and expansion [11]. Exogenous JA was found to be essential for yam tuberization, and induced an increase in the number of tubers in vitro and in vivo [12, 13]. However, fundamental knowledges of endogenous metabolic networks are poor in tuber expansion.

The induction and growth of microtubers in vitro were controlled by nutrients, and sucrose concentration was the most crucial factor affecting tuberization and frequency of proliferation in yam [7, 14]. Yam tuber morphology was significantly correlated with nutrient accumulation and enzymatic activity. Sucrose, soluble sugars, and proteins increased significantly during tuber expansion stage, then subsequently decreased at maturity stage. Starch content increased throughout tuber morphogenesis, and sucrose synthase, sucrose phosphate synthase, and AGPase were significantly correlated with nutrient accumulation [15]. Although many DNA molecular markers have been used to uncover the genetic diversity and relationship among yam germplasms [16–18], little is known about specific genes involved in tuber morphogenesis. The sucrose synthase 4 and sucrose-phosphate synthase 1 were associated with the earliest stages of starch biosynthesis and storage; a SCARECROW-LIKE gene was involved in the formation of adventitious roots [19]. PE2.1 and PE53 are the members of pectinesterase (PE) superfamily, which may be involved in the regulation of starch and sucrose metabolism and signaling pathways. Therefore, they may play an essential role in microtuber formation [20]. Tuber morphogenesis is a complex biological process involving many specific genes and proteins, especially at yam tuber expansion stage. Transcriptome techniques can efficiently find and detect these genes and proteins. Potato is a tuber crop. Several transcriptome analyses revealed that numerous genes are regulated in early stages of stolon-to-tuber transitions, or tuberization by nutrients, photoperiodic conditions, exogenous hormones, and stress in potato tuber [21–24]. Former transcriptomic study revealed that some putative genes were involved in dioscin biosynthesis [25], along with this chalcone isomerase (CHS), flavanone 3-hydroxylase (F3H), flavonoid 3′-monooxygenase (F3’H), dihydroflavonol 4-reductase (DFR), leucoanthocyanidin dioxygenase (LDOX), and flavonol 3-O-glucosyltransferase (UF3GT) were significantly expressed in flavonoid biosynthesis [26]. However, there are no reports of transcriptome study on tuber expansion.

microRNAs (miRNAs) are small, endogenous, non-coding RNAs that have essential functions in many biological processes, such as the regulations of growth and development, stress response, and metabolism. Many studies have shown that miRNAs play essential roles in root and tuber formation or development [27–29]. miR165/166 regulated root growth by determining the fate of root cells in *Arabidopsis* combined with phytohormone crosstalk, by negatively regulating its target genes auxin response factor ARF10, ARF16 and ARF17 [30]. miRNA172 and miR156 were involved in tuberization process, either as a component or a regulator of long-distance gibberellin signaling pathways [31, 32]. Potato specific miRNA193, miRNA152, and conserved miR172–1, miRNA172–5 showed significant expression during developmental stages of tuberization [28]. However many studies have found that miRNAs are involved in tuber and root development, the miRNA-mediated regulatory network during tuber expansion is still unclear.

Although whole-genome sequencing of the heterozygous diploid Guinea yam (*D. rotundata*) had been performed for sex determination [33], a detailed comparative mRNA and miRNA analysis during yam tuber expansion stage need to be detected. In this study, to identify and analyze the global gene and miRNA expression dataset in tuber expansion, six libraries prepared from *D. opposita* (Chinese yam) cv. Guihuai 16 tuber of initiation stage (GH16_I) and expansion stage (GH16_E) were sequenced by using a BGISEQ-500 platform. Furthermore, the association analysis between mRNA and miRNA expression was done, and the elucidation of the regulatory relationship of miRNA and their corresponding mRNA targets was studied for understanding the expansion of tuber.

## Results

### Overview of RNA-Seq dynamics and small RNA sequencing

**Fig.1:**
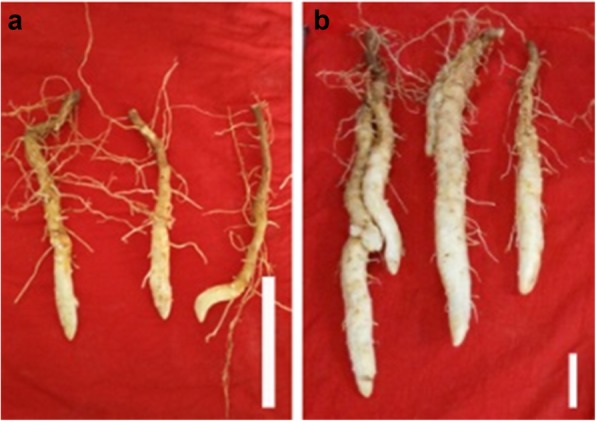
A picture of Guihuai 16 (*D. opposita*) tuber at different developmental stages. Samples were collected from field-grown cultivar Guihuai 16 (*D. opposita*) during its initiation and expansion stages. **a**: Initiation stage, **b**: Expansion stage, white bar is 5 cm

**Fig. 2:**
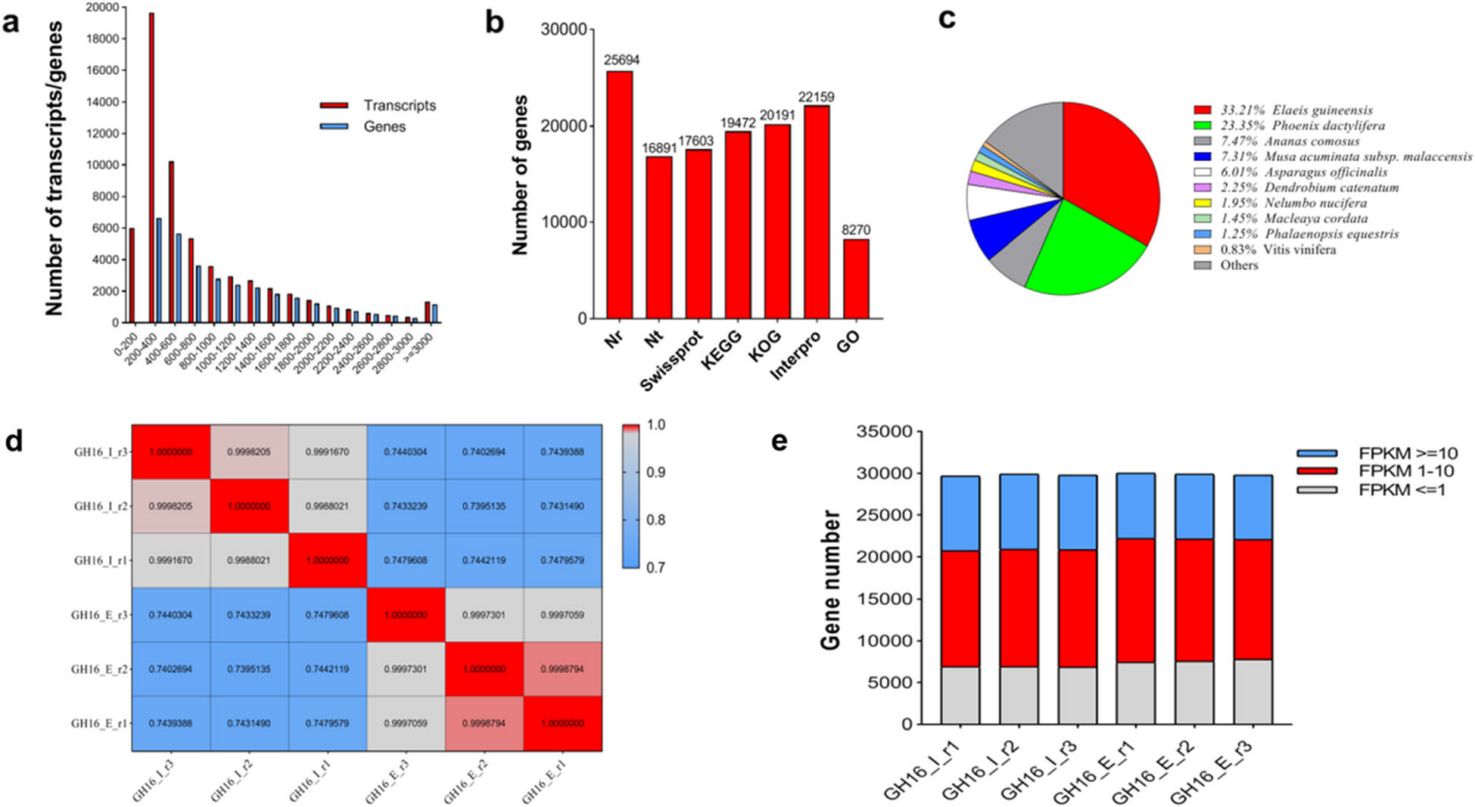
The annotation of Guihuai16 (*D. opposita*) tuber assembled transcriptome and gene expression profiling. **a** Length distribution of assembled cultivar Guihuai 16 (*D*. *opposite*) transcripts and genes, the abscissa represents the length. **b** Number of genes aligned to different databases. **c** Distribution of species aligned by assembled cultivar Guihuai 16 (*D. opposita*) tuber genes. **d** Correlation analysis between samples replicates. **e** Distribution of gene number expression concentration in different FPKM intervals of each mRNA libraries, gray, red and blue represents three situations of FPKM (FPKM<=1, FPKM 1–10, FPKM> = 10), respectively

To identify the regulation of mRNA and miRNAs co-regulatory network during tuber expansion, the RNA-Seq and small RNA were examined during tuber initiation stage (GH16_I) and expansion stage (GH16_E) (Fig. 1). Meanwhile, transcriptome library was constructed from a pool of mixed RNA consisting of initiation and expansion stages in order to construct RNA-Seq and small RNA (named Total_1). Approximately 74.71 Mb original data in total were gained from BGISEQ-500 platform at BGI-Shenzhen (Table 1). After filtering low-quality reads and adaptor sequences, 6.67 Gb clean reads were obtained and processed by de novo analysis using Trinity software. The assembly produced a total of 54,781 transcripts. Then, Tgicl software was used on transcripts to remove abundance, and 32,207 genes were gained. The N50 statistic was 1508, which meant that more than 50% of the genes were longer than 1508 bp. The length distribution of all the assembled yam genes shown in Fig. 2a, which indicated that 7.91% of the complete transcripts and 13.00% of the total genes were longer than 2000 bp.

**Table 1:** Statistic analysis of clean reads for mRNA in tuber initiation and expansion stages in yam

Sample	Total_1	GH16_I_r1	GH16_I_r2	GH16_I_r3	GH16_E_r1	GH16_E_r2	GH16_E_r3	Sum
Total Raw Reads (Mb)	74.71	66.43	66.42	66.52	66.43	66.43	66.35	–
Total Clean Bases (Gb)	6.67	6.57	6.57	6.58	6.56	6.57	6.56	–
Clean Reads Q20(%)	96.4	98.63	98.56	98.54	98.52	98.5	98.57	–
Clean Reads Q30(%)	88.22	93.12	92.86	92.78	92.67	92.63	92.95	–
Clean Reads Ratio(%)	89.34	98.91	98.92	98.96	98.7	98.87	98.9	–
Total Mapped Reads(%)	–	83.07	83.34	83.17	82.05	81.77	82.03	–
Total Expressed Genes	–	29,658	29,905	29,839	30,012	29,893	29,811	32,026
Total trinity Transcripts	54,781							
Total Tgicl Genes	32,207							
GeneGC(%)	44.63							
GeneN50	1508

A total of 32,207 genes were functionally annotated with 7 functional database.

(NR, NT, GO, KOG, KEGG, SwissProt, and InterPro), 25,694 (79.78%), 16,891 (52.45%), 17,603 (54.66%), 19,472 (60.46%), 20,191 (62.69%), 22,159 (68.80%), 8270 (25.68%) reads were annotated functionally respectively (Fig. 2b). 13,566 genes were commonly annotated in NR, KOG, KEGG, SwissProt and InterPro databases. Based on the functional annotation results of NR database, the proportions of different species in the notes of genes were calculated, 8533 (33.21%), 5999 (23.35%), 1920 (7.47%) and 1879 (7.31%) genes were aligned to *Elaeis guineensis*, *Phoenix dactylifera*, *Ananas comosus*, *Musa acuminata subsp and Malaccensis* (Fig. 2c). Similar species distributions were also observed for yam tuber in previous research [25], 8229 (16.2%) genes in *D. zingiberensi*s had the most hits from *Elaeis guineensis*, followed by *Phoenix dactylifera* (6857, 13.5%), *Musa acuminate* (2692, 5.3%).

After filtering low-quality reads and adaptor sequences, 6.57 Gb, 6.57 Gb, 6.58 Gb, 6.56 Gb, 6.57Gb, and 6.56Gb clean reads were obtained in six RNA-Seq analysis libraries (initiation stage named GH16_I, expansion stage named GH16_E) respectively, and a total of 32,026 expressed genes were detected (Table 1). The average mapping rate of transcriptome library (named Total_1) was 82.57%. A heat map cluster showed good correlations among replicates which indicated high repeatability of the data (Fig. 2d). In order to show the number of genes in different FPKM intervals of each mRNA libraries more intuitively, three situations of FPKM (FPKM<=1, FPKM 1–10, FPKM> = 10) were counted the number of genes (Fig. 2e), indicating that most genes were expressed in the FPKM 1–10 ranges in the libraries. Genes with expression levels > 5 FPKM were retained for statistical analysis.

Furthermore, the corresponding six small RNA libraries at the three time-points were also constructed for deep sequencing. Initially, a total of 170,957,171 reads were generated (Table 2). After filtering low-quality reads and adaptor sequences, 157,958,048 clean reads longer than 18 nt for six libraries with an average of 26.32 M clean reads were obtained, and length distribution of clean reads showed that the classes of sRNA were 21-24 nt (Additional file 1: Figure S1). Subsequently, 6,388,211 (25.11%), 5,872,589 (22.36%), 6,086,348 (22.98%) reads in tuber initiation stage and 4,593,044 (17.48%), 5,032,588 (18.58%), 4,642,869 (17.58%) reads in tuber expansion stage were mapped to sRNA database (rRNA, tRNA, snRNA and snoRNA), respectively.

**Table 2:** Statistic analysis of clean reads for small RNA sequencing in tuber initiation and expansion stages in yam

Sample name	Tota reads	Clean reads	Mapped reads	Known miRNA	Novel miRNA	Total miRNA
GH16_I_r1	27,687,839	25,438,600	6,388,211	18	50	68
GH16_I_r2	28,663,585	26,268,556	5,872,589	19	47	66
GH16_I_r3	28,610,515	26,482,287	6,086,348	21	49	70
GH16_E_r1	28,256,295	26,276,062	4,593,044	20	49	69
GH16_E_r2	29,343,139	27,085,820	5,032,588	20	49	69
GH16_E_r3	28,395,798	26,406,723	4,642,869	22	50	72
Sum	170,957,171	157,958,048				

### Differentially expressed genes annotation by GO term and KEGG pathway

To identify differentially regulated genes in tuber expansion stage, DESeq software was used to compare the changes of gene expression between initiation and expansion stages. Among them, 5723 genes were up-regulated, 8515 genes were down-regulated, respectively, and it were differentially expressed in expansion stage (GH16_E), compared to initiation stage (GH16_I) (Additional file 2: Table S1).

**Fig. 3:**
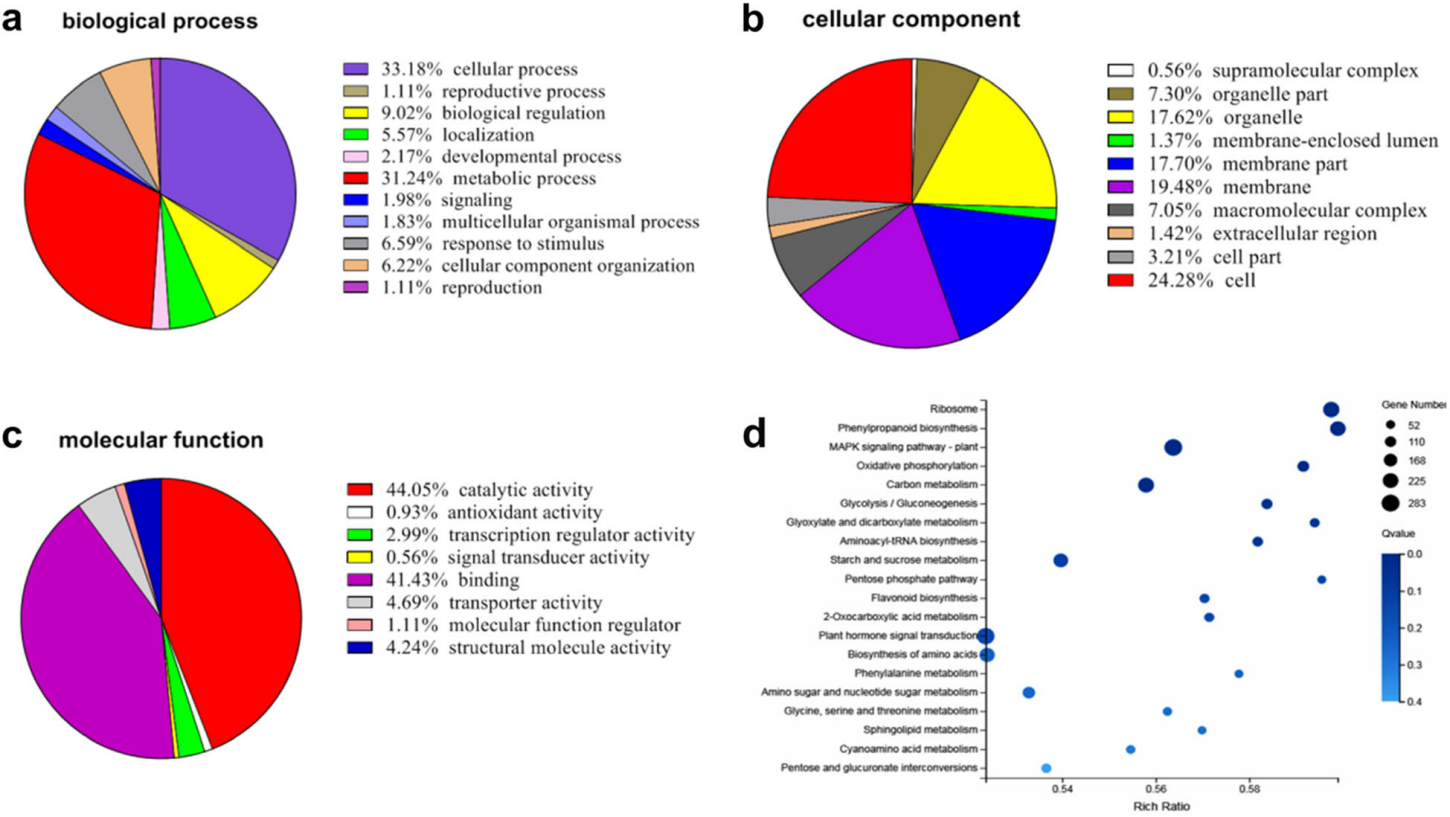
Gene Ontology and KEGG pathway annotation of Guihuai16 (*D. opposita*) tuber assembled genes. **a**, **b** and **c** represent the biological process, cellular component, and molecular function, respectively. **d** Top 20 significant KEGG pathways

For better comprehension of DEGs functions, 44 GO categories were identified. For biological processes, DEGs associated with cellular process (33%), metabolic process (31%), and biological regulation (9%) were enriched during expansion stage (Fig. 3a). For cellular component, 10 GO categories were enriched in DEGs, including cell (24%), membrane (19%), membrane part (18%), and organelle (18%) (Fig. 3b). The molecular functions of the DEGs were mainly associated with catalytic activity (44%), binding (41%), transporter activity (5%), structural molecular activity (4%) (Fig. 3c). Among the significant GO term analysis, 15 genes were enriched in cell wall polysaccharide metabolic process (GO:0010383), 15 genes were involved in hemicellulose metabolic process (GO:0010410), and 13 genes were related to xyloglucan metabolic process (GO:0010411) related to cell wall formation during expansion stage (Table 3). Besides, the results also revealed several significant expression genes involved in tissue development, root morphogenesis, root system development, and root development (Table 4).

**Table 3:** GO enrichment analysis of DEGs

Type	ID	Term	Gene Number	Rich Ratio	*P* value
Molecular function	GO:0005198	Structural molecule activity	183	0.62	1.83867E-07
	GO:0003735	Structural constituent of ribosome	143	0.61	1.71036E-05
	GO:0003700	DNA binding transcription factor activity	116	0.59	0.001099451
Cellular component	GO:0005576	Extracellular region	91	0.70	2.22612E-07
	GO:0043228	Non-membrane-bounded organelle	285	0.58	1.68443E-06
	GO:0043232	Intracellular non-membrane-bounded organelle	285	0.58	1.68443E-06
	GO:0005840	Ribosome	157	0.61	1.90586E-05
	GO:0005618	Cell wall	49	0.72	4.36888E-05
	GO:0030312	External encapsulating structure	49	0.72	4.36888E-05
	GO:0048046	Apoplast	32	0.78	0.000075892
	GO:0022625	Cytosolic large ribosomal subunit	22	0.81	0.000355453
Biological process	GO:0044262	Cellular carbohydrate metabolic process	77	0.69	1.68372E-05
	GO:0005975	Carbohydrate metabolic process	171	0.60	0.00025216
	GO:0044264	Cellular polysaccharide metabolic process	53	0.70	0.000285538
	GO:0045786	Negative regulation of cell cycle	11	1.00	0.000452873
	GO:0006073	Cellular glucan metabolic process	50	0.69	0.000495823
	GO:0010383	Cell wall polysaccharide metabolic process	15	0.88	0.001069447
	GO:0010410	Hemicellulose metabolic process	15	0.88	0.001069447
	GO:0010411	Xyloglucan metabolic process	13	0.93	0.000841262

**Table 4:** Functional classification and pathway assignment of differentially expressed DEG by GO in expansion stage

Tissue development [GO:0009888]	Gene ID	log2(GH16_E/GH16_I)
Actin-related protein 3	CL1179.Contig2_Total_1	−2.00
Anaphase-promoting complex subunit 10	CL1997.Contig4_Total_1	1.82
Alpha-tubulin	CL28.Contig3_Total_1	−5.08
Tubulin alpha chain	CL3054.Contig1_Total_1	−1.81
Glutamine synthetase nodule isozyme	Unigene128_Total_1	−4.98
Phosphoenolpyruvate carboxylase 2	Unigene13453_Total_1	−1.23
Phosphoenolpyruvate carboxylase 2	Unigene13455_Total_1	1.32
Anaphase-promoting complex subunit 10	Unigene18039_Total_1	− 1.15
Anaphase-promoting complex subunit 6	Unigene2600_Total_1	−1.06
Actin-related protein 2	Unigene4771_Total_1	−1.10
ATPase ASNA1 homolog	Unigene5972_Total_1	−1.32
Homeobox protein knotted-1-like 3	Unigene6778_Total_1	3.48
Root morphogenesi, root system development, root development [GO:0009888,GO:0022622,GO:0048364]		
Cytoplasmic tRNA 2-thiolation protein 2	CL1237.Contig1_Total_1	−1.02
Cytoplasmic tRNA 2-thiolation protein 2	CL1237.Contig2_Total_1	−2.28
Mediator of RNA polymerase II transcription subunit 32	CL2787.Contig2_Total_1	2.02
Succinate dehydrogenase assembly factor 2	CL3034.Contig1_Total_1	−1.04
Guanine nucleotide-binding protein	Unigene18752_Total_1	−3.18
Enhanced ethylene response protein 5	Unigene2193_Total_1	−1.75
ATPase ASNA1	Unigene5972_Total_1	−1.32

KEGG is a signal pathway database with vibrant signal pathway map, 20 pathways were identified during yam tuber expansion stage. Interestingly, KEGG pathway analysis showed that plant hormone signal transduction (ko04075), biosynthesis of amino acids (ko01230) were enriched with DEGs during expansion stage (Fig. 3d). Other pathways such as MAPK signaling pathway (ko04016), starch and sucrose metabolism (ko00500), and carbon metabolism (ko01200) were also identified as involving 283, 204, and 236 DEGs, respectively. The metabolic pathways may be closely related to the development of tuber expansion and bioactive compound synthesis.

### Comprehensive analysis of differentially expressed genes in expansion stage

Compared with initiation stage, there were a large number of DEGs in tuber expansion stage using NR, GO, and KEGG annotation. Signal transduction, cell wall, cell division, starch, and sucrose metabolism were selected for profiling during the expansion of yam tuber.

### Hormone signal

A total of 242 DEGs were identified to be highly similar to many plant hormone signal pathways, including 131 down-regulated and 111 up-regulated DEGs in expansion stage (Additional file 2: Table S1). Interestingly, most plant hormone-related genes in GA, IAA, and ABA signal pathways were discovered during expansion stage.

In auxin transduction pathway, the transcriptional level of auxin influx carrier /auxin-responsive protein IAA (AUX/IAA) and small auxin up RNA (SAUR) were significantly down-regulated during the expansion stage, while auxin-responsive GH3 gene family (GH3) was up-regulated. In contrast, two auxin response factor ARFs (CL2135.Contig1_Total_1, and Unigene5660_Total_1) were shown high expression level during expansion stage, while other two ARFs (CL2887.Contig2_Total_1, Unigene5486_Total_1) were low expression level during expansion stage (Additional file 3: Table S2).

In gibberellin transduction pathway, the expression of gibberellin receptor GID2 was low expression during expansion stage. In contrast, DELLA proteins were highly expressed during the expansion stage. Meanwhile, protein phosphatase 2C(PP2C) was highly expressed during expansion stage.

### MAPK and calcium signaling

Regulation of genes related to MAPK and calcium signaling during the expansion stage were also investigated. Six mitogen-activated protein kinases (MAPK) genes were up-regulated during expansion stage, while MPK6 and MPK8 were down-regulated. In summary, 48 DETs were homologous with calcium signal-related genes (Additional file 2: Table S1), including calcium-dependent protein kinases (CDPKs), calcium-binding proteins (CBPs), and calreticulin (CBL). It is worth noticing that CBLs were down-regulated during expansion stage (Additional file 3: Table S2).

### Cell wall and cell cycle

A total of 98 transcripts homologous to the genes associated with cell wall and cell cycle were observed as differentially regulated during expansion stage (Additional file 2: Table S1), including xyloglucan endotransglucosylase/hydrolase (XTH), expansin, extension, cyclin-dependent kinases (CKS), cell division protease (ftsHs), cell division cycle 5-like protein (CDC5), cell division control protein (CDC), cyclin-dependent kinases (CDKs), and cyclin-dependent kinase inhibitor (CDKIs). All of the expansin, extension, cell wall synthesis, and CKS genes were down-regulated during expansion stage. Meanwhile, most of the cell cytoskeleton and XTH were down-regulated during expansion stage in yam (Additional file 3: Table S2).

### Starch and sucrose metabolism

The major constituents of starch and sucrose metabolism during expansion stage are sucrose synthase genes (SuSy), sucrose phosphate synthase genes (SPS), starch synthase (SS), and invertase genes (INV) (Additional file 2: Table S1). Among them, SuSy were down-regulated during expansion stage. Interestingly, dioscorins, the major storage proteins in yam tubers, were significantly up-regulated during the expansion stage (Additional file 3: Table S2). These results indicated that many functional genes were involved in expansion stage of yam tuber.

### Transcription factor

**Fig. 4:**
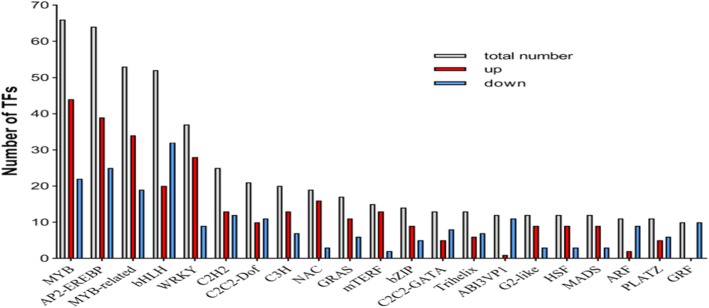
The numbers of up-regulated and down-regulated transcription factors during expansion stage

**Fig. 5:**
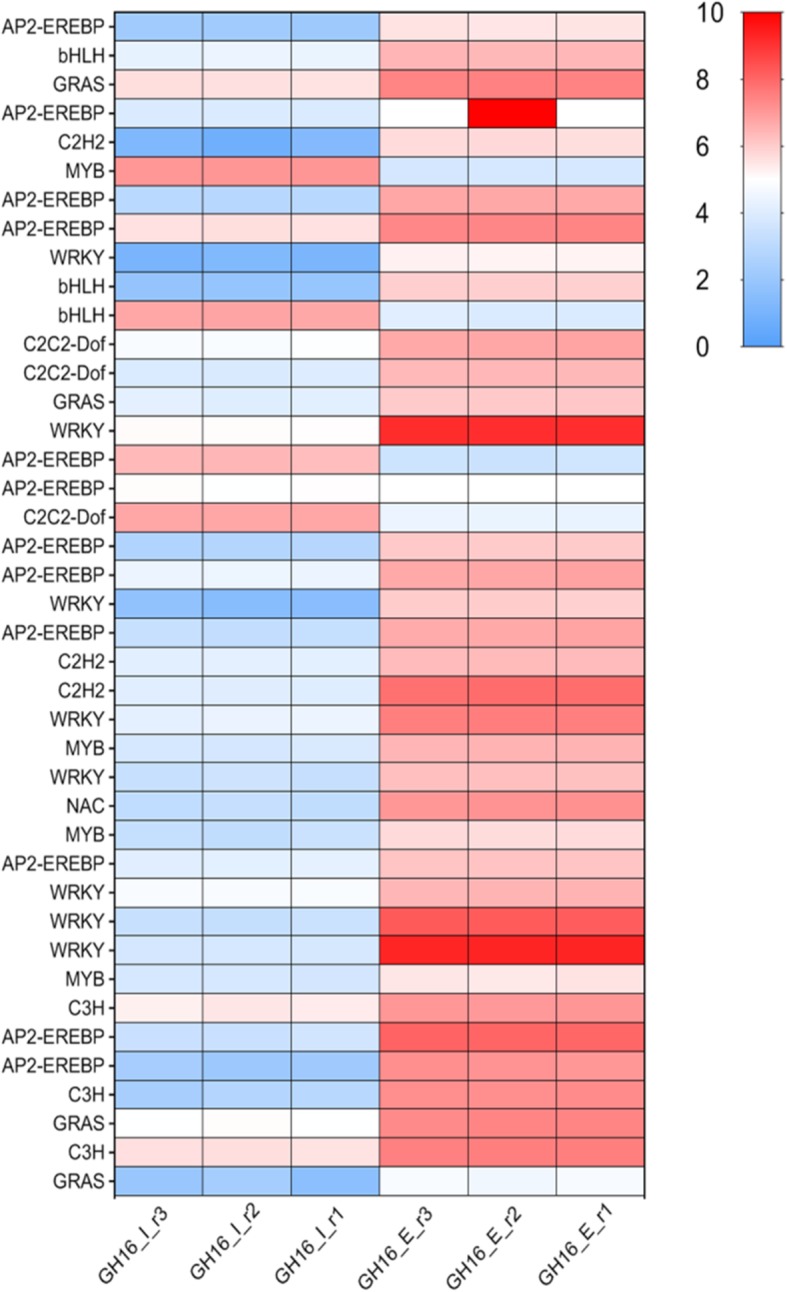
Heat map representing the levels of highly expressed transcription factors. The color scale on the right represents log-transformed FPKM value. Every row shows a different TF gene. Red, white, and blue indicate slow, medium and high levels of mRNA expression, respectively

A total of 541 TF-encoding genes belonging to 48 TF families were differentially expressed during expansion stage, MYB, MYB-related, and AP2-EREP were enriched (Fig. 4). 286 TF encoding genes were up-regulated and 255 TF encoding genes were down-regulated, respectively (Additional file 4: Table S3). The most abundant TF gene families with the highest number of expressions during expansion stage were depicted by heat map (Fig. 5). Moreover, these genes were involved in circadian rhythm pathway, starch and sucrose metabolism pathway, and GA pathway by KEGG analysis respectively.

### Detection of known and novel miRNAs expressed in tuber initiation and expansion stages

The investigation of both known miRNA and novel putative miRNAs was performed by miRDeep2 program. This program combined the position and frequency of small RNAs with the secondary structure of miRNA precursor to provide novel miRNA that can accurately be in the tubers. To compare miRNA expression in six libraries, the number of clean reads was used as background for normalization, and transcripts per million reads (TPM) was used to present the expression levels of miRNAs. Data analysis showed that there were 22 known miRNAs (21 and 20 in tuber initiation and expansion stage, respectively) and 50 novel miRNAs in yam tuber (Additional file 5: Table S4) and 68, 66, 70 total miRNAs were detected in tuber initiation stage (GH16_I), and 69, 69, 72 total miRNAs were detected in tuber expansion stage (GH16_E), respectively (Table 2). Distribution of normalized miRNAs expression showed that approximately 75–81% of the total detected miRNA expression exceeded 10 TPM in six libraries (Additional file 5: Table S4).

Further analysis revealed that 22 known miRNAs belonging to 10 miRNA families, miRNA168, miRNA396, and novel miRNA160 were the most extensively represented families. All miRNAs were analyzed to detect differential miRNA expressions (DEMs). The results showed that miRNAs expression was dynamically regulated during expansion stages. A total of 44 differentially expressed miRNAs were identified including 11 known and 33 novel miRNAs; it showed that 40 miRNAs were down-regulated, and 4 miRNAs were up-regulated during expansion stage (Table 5). Interestingly, miR160 family (miRNA160, miRNA160a-5p, miRNA160b-1, miRNA160h-1) and miR535a_1 were down-regulated during expansion stage, miRNA396b and miRNA168 were up-regulated compared to other miRNA families (Table 5).

**Table 5:** Differentially expressed miRNAs involved in expansion stage

miRNA id	Read count (GH16_I)	Read count (GH16_E)	Expression (GH16_I)	Expression (GH16_E)	log2Ratio (GH16_E/GH16_I)	P value
novel_mir43	54,974	33,041	3682.20	1731.72	− 1.97	0.00000
novel_mir35	52,437	32,873	3515.56	1728.51	−1.91	0.00000
miR160a-5p	18,032	121	1171.81	6.42	−8.45	0.00000
miR168a-5p	55,653	59,949	3708.90	3153.82	−1.13	0.00000
miR168	20,208	106,873	1334.10	5618.38	1.17	0.00000
novel_mir1	16,275	7760	1085.59	407.58	−2.30	0.00000
miR396b	1097	21,915	72.84	1176.08	3.09	0.00000
miR535a_1	24,978	25,221	1654.31	1315.07	−1.22	0.00000
miR396a-5p	12,295	7317	816.34	374.44	−1.98	0.00000
novel_mir33	6408	4269	425.49	224.55	−1.82	0.00000
novel_mir15	9666	10,433	642.81	549.12	−1.12	0.00000
novel_mir11	4852	3769	323.07	199.13	−1.60	0.00000
novel_mir25	5833	6318	386.43	333.34	−1.12	0.00000
novel_mir32	3196	2381	212.72	125.72	−1.66	0.00000
novel_mir5	2039	1094	135.31	58.03	−2.13	0.00000
novel_mir50	2777	2281	183.85	120.27	−1.52	0.00000
novel_mir10	3197	3009	213.02	158.49	−1.32	0.00000
novel_mir44	3381	3299	224.65	173.72	−1.27	0.00000
novel_mir36	1859	1246	124.13	65.29	−1.81	0.00000
novel_mir31	1632	1049	108.56	55.06	−1.87	0.00000
novel_mir18	1701	1305	114.05	68.55	−1.62	0.00000
novel_mir19	1491	1071	99.15	56.35	−1.71	0.00000
novel_mir12	1666	1308	111.28	69.05	−1.58	0.00000
novel_mir45	1478	1093	99.12	57.63	−1.67	0.00000
miR160	926	451	62.61	24.59	−2.27	0.00000
novel_mir48	1107	690	74.52	36.21	−1.92	0.00000
novel_mir26	1488	1201	99.79	63.01	−1.54	0.00000
miR160b_1	463	30	30.27	1.62	−5.18	0.00000
novel_mir46	1268	926	83.82	48.75	−1.69	0.00000
novel_mir42	694	275	46.22	14.50	−2.57	0.00000
novel_mir49	676	265	45.07	13.82	−2.59	0.00000
novel_mir24	1045	779	69.79	40.94	−1.66	0.00000
novel_mir23	377	109	25.16	5.68	−3.03	0.00000
miR168a-3p	1330	1489	87.83	78.66	−1.07	0.00000
novel_mir20	374	2206	24.76	116.77	1.33	0.00000
novel_mir30	964	999	63.39	52.50	−1.18	0.00000
novel_mir37	173	45	11.52	2.37	−3.18	0.00000
novel_mir47	90	10	6.03	0.50	−4.40	0.00000
novel_mir4	394	447	25.80	23.70	−1.05	0.00000
novel_mir34	10	200	0.68	10.70	3.09	0.00000
novel_mir8	197	207	13.12	10.72	−1.16	0.00000
miR168_1	30	5	1.98	0.26	−3.82	0.00000
novel_mir9	123	142	8.24	7.48	−1.03	0.00000
miR160h_1	7	0	0.46	0.00	−5.04	0.00041

### Identification of target genes of differentially expressed miRNA in tuber expansion compared with initiation stage

**Fig. 6:**
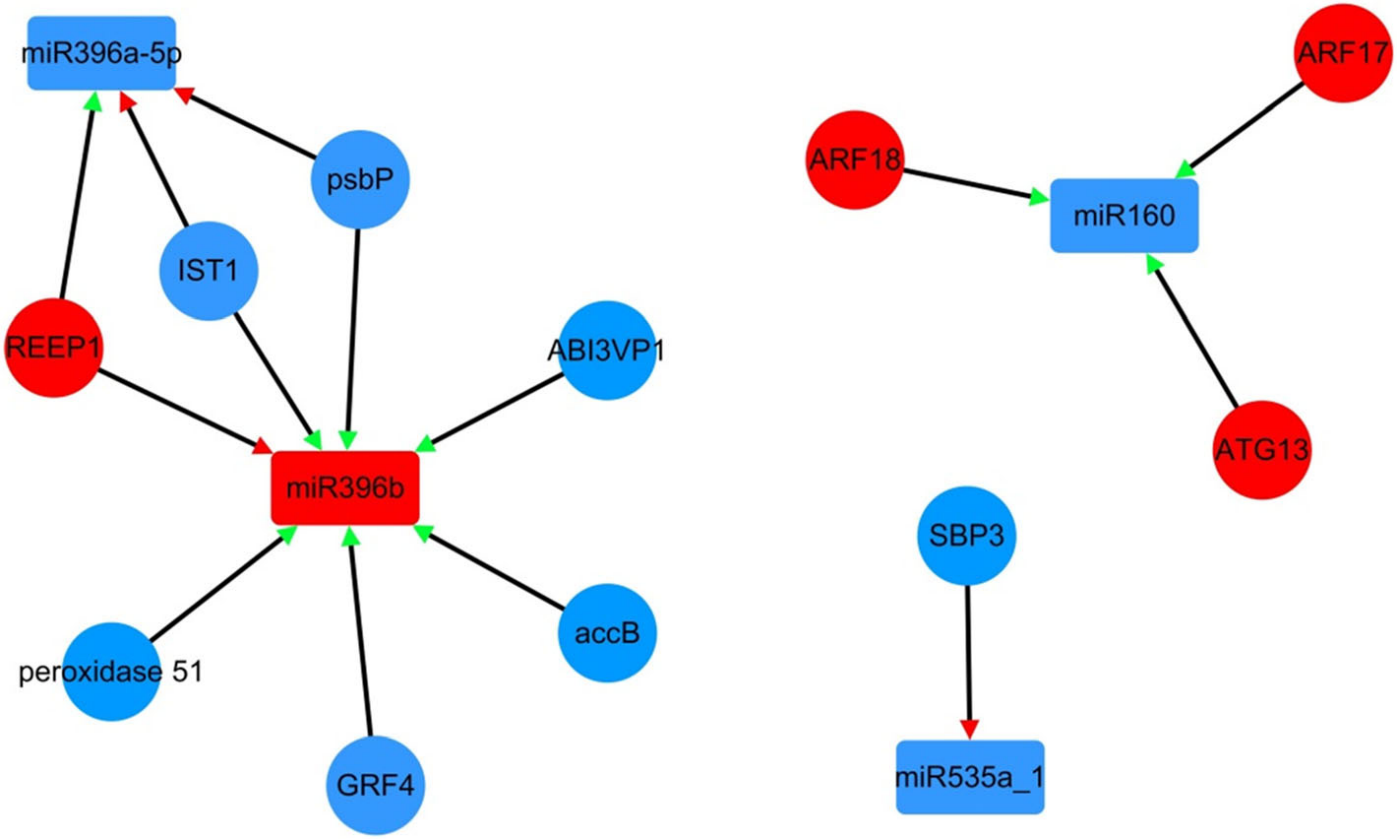
Regulatory network from the integrated analysis of miRNA-mRNA data. Co-expressed miRNA-mRNA interactions visualized as a network using Cytoscape. Triangle represents the expression type of miRNA (red triangle represents positive correlation, green triangle represents negative correlation in each miRNA-mRNA), red represents up-regulation and blue represents down-regulation in network, the circle represents mRNA, the rectangle represents miRNA

The miRNA regulates target mRNA through translational repression or mRNA degradation. To identify the correlation between the expression of DEMs and DEGs, a total of 11 DEMs were putatively targeted to 34 DEGs (Additional file 6: Table S5). Notably, 4 known miRNAs and 11genes formed 14 miRNA-target mRNA pairs with co-expressed expression in expansion stage (Fig. 6). Furthermore, based on GO and KEGG analysis of the targets, it was showed that differentially expressed miRNAs were involved in multiple pathways. Some of target genes of miRNA were annotated as transcription factors, such as two auxin response factor ARFs (ARF17 and ARF18, targeted by miRNA160), growth-regulating factor 4 (GRF, targeted by miRNA396b), B3 domain-containing protein (ABI3VP1, targeted by miRNA396b), and squamosa promoter-binding-like protein 3 (SBP3, targeted by miR535a_1).

On the other hand, some target genes were revealed to be involved in transport and catabolism processes, such as autophagy-related protein 13b (ATG13, targeted by miRNA160), IST1-like protein (IST1, targeted by miR396a-5p and miR396b). Finally, some genes were annotated as other functional proteins, such as receptor expression-enhancing protein1 (REEP1, targeted by miR396a-5p and miR396b), photosystem II oxygen-evolving enhancer protein 2 (psbP, targeted by miR396a-5p and miR396b), and acetyl-CoA carboxylase biotin carboxyl carrier protein (accB, targeted by miR396b).

### Validation of the DEGs and DEMs data using RT-qPCR

**Fig. 7:**
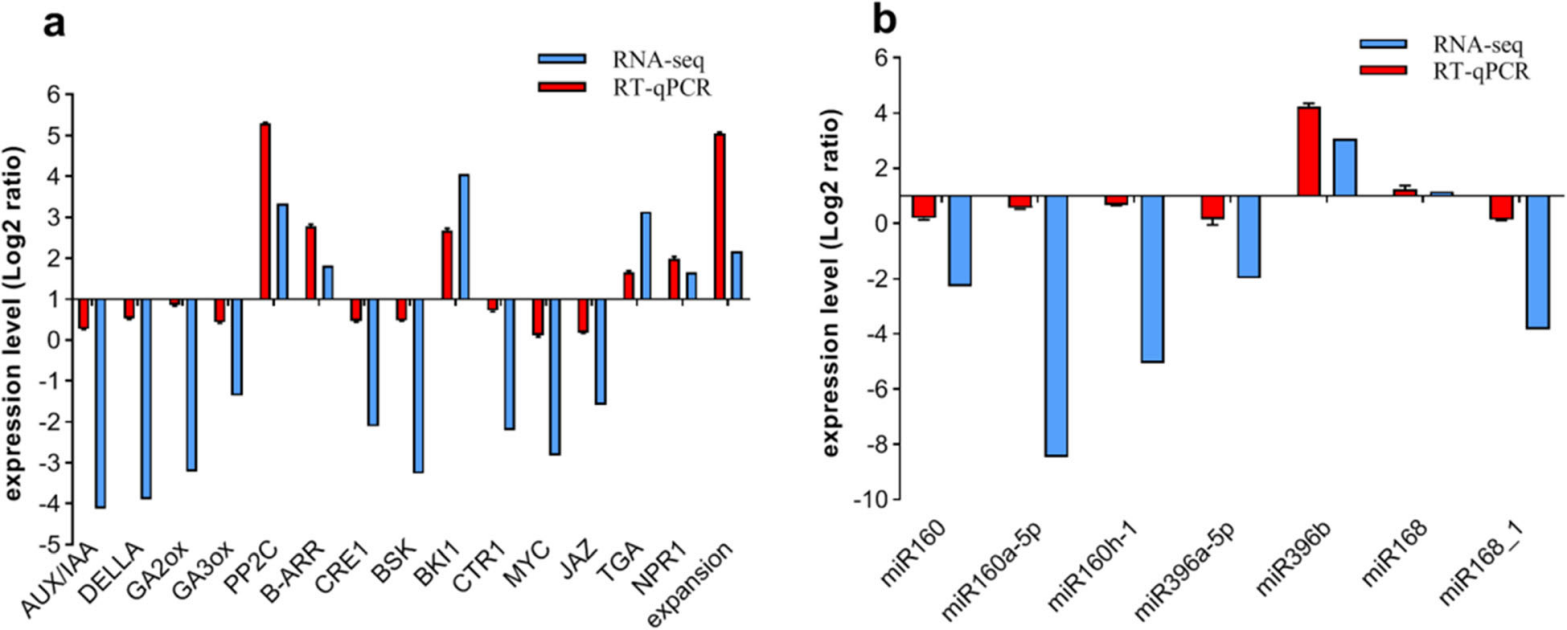
Verification of (**a**) DEGs and (**b**) miRNAs by qRT-PCR

To identify the accuracy and reliability of RNA-Seq and miRNA data, RT-qPCR was used to measure the expressions of some DEGs and DEMs, including 15 mRNAs, and 7 miRNAs, specific primers were designed (Additional files 7 and 8: Tables S6 and S7). All mRNA and miRNA expressions have confirmed the accuracy of RNA-Seq and small RNA data. Overall, these results showed that 15 mRNAs showed similar expression patterns compared to DEGs analysis (Fig. 7a), and 7 miRNAs also showed very similar patterns compared to DEMs analysis (Fig. 7b). These results indicated that the RNA-Seq and small RNA data were reliable.

## Discussion

In tuber plants, tuber development is based on successive gene expression. Recently, the gene expression analysis of tuber development has been studied by transcriptome analysis, such as *Solanum tuberosum*, *Miscanthus lutarioriparius*, *Raphanus sativus*, *Nelumbo nucifera* and *Aconitum heterophyllum* [28, 34–37]. Yam is one of the most commercial stem tuber crops. Yam tuber expansion stage is an attractive theoretical model for studying the development of underground organs because it is the primary storage organ as nutrients reservoir. Therefore, the understanding of candidate genes involved in tuber expansion stage is supreme importance. In this study, 14,238 genes were identified that have shown significant differential expression during expansion stage.

### Multiple signaling pathways regulation

KEGG enrichment analysis revealed that a large number of genes were involved in pathways related to MAPK signaling pathway, plant hormone signal transduction, starch and sucrose metabolism, and carbon metabolism (Fig. 3d). These genes are involved in cell wall biosynthesis, cell proliferation, cell expansion, nutrient accumulation, primary metabolism, and hormone signal transduction [34, 38, 39]. Cellular processes are triggered by specific stimuli and hormones that are involved in a series of signaling pathways. Calcium transduction pathway is a primary essential signaling transduction category required for normal growth and development of plants. As a second messenger, it plays an important role in many fundamental cellular processes such as cytoplasmic streaming, thigmotropism, gravitropism, cell division, cell differentiation, photomorphogenesis, plant defense, and various stress responses [40–42]. A potato Ca^2+^-dependent protein kinase, *StCDPK1*, which was reported to be expressed during tuberization, possibly be involved in potato tuberization through transcriptional activation of certain genes [40, 43]. In addition, *StCDPK1* was affected by JA, ABA, GA treatment [43–45], suggested that CDPK possibly be a mediator of hormone-to-tuber stimulation.

Moreover, majority of studies have demonstrated that mRNA abundance of CBL, CBP, and GaM were affected by cell division during swelling of potato tubers [40, 43, 46]. In this study, expression of calcium signaling-related genes, including CDPK and CBPs, increased during tuber expansion stage. Interesting, CBLs were down-regulated (Additional file 3: Table S2). The role of CBL in root development is characterized by a robust gene expression during cell proliferation in the root, and its expression in root is regulated by auxin and cytokinin [47, 48]. These results demonstrated that calcium signal is regulated during tuber expansion stage. Besides, MAPK signal pathways are known to play a central role in cell proliferation, differentiation and hormones, but it is not known to be involved in tuber or root formation [49]. In this study, MAPK signal-related genes were up-regulated during expansion stage, and it means that MAPK signal pathways take part in yam tuber expansion. Cell number and cell size are two key determinants of plant and organ size, while involved in cell division, cell expansion and cell cycle [50, 51]. In this study, XTH, expansin, extension, CKS, ftsHs, CDC5, CDC, CDKs, and CDKIs involved in cell division, cell expansion and cell cycles were differentially regulated during expansion stage. These differentially regulated genes involved in cell wall and cell cycle metabolism were identified in *Arabidopsis*, *Rehmannia glutinosa*, and radish [35, 36, 52].

### Transcription factors regulation

Transcription factors have been identified to play an essential role in the regulation of plant growth, development, and secondary metabolism. Most transcription factors have been identified to play critical roles in organogenesis, including MADS, bHLH, and GRAS. In sweet potato, MADS were preferentially expressed in root at initiation of tuberization, and in the vascular cambium region where the active cell proliferation regulated by jasmonic acid, cytokinins and stress response [53, 54]. In *Arabidopsis*, bHLH family, such as PIF3, PIF4, and PIF5 are key regulators for cell elongation during seedling development, antagonistically regulated by light and gibberellins [55]. GRAS transcription factor family specifically regulated the initiation of axillary meristem in *Arabidopsis*, such as SCARECROW (SCR) is expressed in cortex/endodermal initial cells in the root system and played a crucial role in regulating the radial organization of the root [56]. DELLA, such as RGA and GAI, controls cell expansion and cell division in hypocotyl, shoot, root, and floral induction [57]. In this study, 12 MADS, 52 bHLH, and 17 GRAS were found to be expressed differently during expansion stage at transcript level, which plays key regulatory functions in cell differentiation, division and expansion (Fig. 4). Zinc finger CCCH domain-containing protein 14 (C3H) has shown to be involved in growth and development, hormone response, and response to biotic and biotic stresses [58]. All data suggest that these transcription factors may be potentially involved in the expansion stage.

### Hormonal signaling regulation

A set of transcriptome evidence suggests that hormone signal pathway-related genes involved in regulating of storage organ formation have been identified in sweet potato, potato, radish, lotus, and carrot, while may be potentially involved in cell division, differentiation, and expansion [1, 35, 37, 59, 60]. In the KEGG pathway annotation, hormone signal pathway was the most enriching one that was involved in eight signal pathways (Fig. 3d). In radish, most Aux/IAA, ARFs, and SAUR genes were abundantly expressed in the root cortex splitting and expansion stage, implying that these transcripts may be involved in cambium cell expansion [35]. Among auxin signal-related genes, Aux/IAA, ARFs were highly expressed in expansion stage in this study, and auxin has been identified to regulate cell division and expansion by altering genes expression [1, 38], suggesting that Aux/IAA, ARFs may offer excellent candidates during expansion stage.

Interestingly, in this study, DELLA genes were up-regulated, while GIDI, like GID2, were down-regulated during expansion stage (Additional file 3: Table S2). DELLA proteins are nuclear transcriptional regulators that repress GA signaling and may restrict plant growth presumably by causing transcriptional reprogramming. Binding of GA to GID1 enhances the interaction between GID1 and DELLA, resulting in rapid degradation of DELLAs via the ubiquitin-proteasome pathway. The GA-GID1-DELLA signal control hypocotyl root elongation by reducing GA levels and DELLA interacts directly with PIF4, PIF3, and SCL to mediate crosstalk between GA and light signals [55, 61]. DELLA is significantly expressed in GA-mediated rhizomes, suggesting that DELLAs can regulate rhizomatic expansion [37]. Besides, GA, IAA, and ethylene affect cell growth in roots via DELLA proteins [57]. Overall, these results indicate that hormone signaling-related genes have a complex regulatory network involved in tuber expansion.

### Starch and sucrose metabolism regulation

Starch and sucrose are considered to be one of the significant carbohydrates source, in which expansion tubers are highly coordinated with starch and sucrose metabolism genes in potato, radish, and lotus identified by transcriptome analysis [22, 35, 37]. In this study, alpha-amylase, beta-amylase, and isoamylase were up-regulated (Additional file 2: Table S1), which is similar to increase in beta-amylase activity in swollen taproot in radish [35], where starch content decreased during the tuber expansion stage [15], and some sucrose metabolism genes were detected during expansion stage, including SuSy, SPS, INV, and invertase inhibitor (Additional file 2: Table S1). Evidence shows that sucrose can be converted to starch in storage root by SuSy and AGPase, which means that they play a vital role in the early stage of radish expansion [35]. The SuSy gene was down-regulated during expansion stage. The results were similar to the taproot expansion stage in radish [35], implying that they may play a significant role in tuber expansion stage of yam. Yam tuber morphology is significantly correlated with sucrose and starch [15]. Therefore, these starch and sucrose metabolism genes are necessary for tuber expansion.

### miRNAs regulation by targeting the potential genes

miRNA mediated gene regulation has been extensively studied in root and tuber development through transcriptional and post-transcriptional levels, which provides a better understanding of molecular regulatory network during tuber expansion stage. In this study, most miRNAs were down-regulated during expansion stage (Table 5). In maize leaves, miR160 was significantly up-regulated in meristem relative to the elongation and mature zones [62]. However, miR160 was down-regulated during yam tuber expansion stage (Table 5). In general, miR160 expression is different in different plants. In this study, some miRNA-mRNA pairs were observed during expansion stage (Fig. 6), miR160 was involved in root cell division and differentiation by regulating auxin response factors (ARFs) affecting root development in *Arabidopsis thaliana* [30]. miRNA160 was down-regulated during expansion stage, auxin response factor ARF18 and ARF17 were up-regulated during expansion stage (Fig. 6), whereas, overexpression of miR160 in transgenic rice not only down-regulated the expressions of ARF10, ARF16, and ARF17, but also inhibited root-cap cells differentiation, lost control of cell division and led to ectopic expansion of the apical stem cell populations [63]. Previous studies have shown that the miRNA160-targeted ARF has been identified to be involved in cell expansion and cell differentiation in radish root and potato tuber [28]. In addition, miRNA396 plays an essential role in root growth and inhibits leaf cell division by UV-B radiation [64, 65]. miR535 is expressed in fruit development [66]. miRNA160, miRNA396, and miR535 may be involved in complex a network of regulating cell division and differentiation during expansion stage.

### Regulatory networks associated with tuber expansion

**Fig.8:**
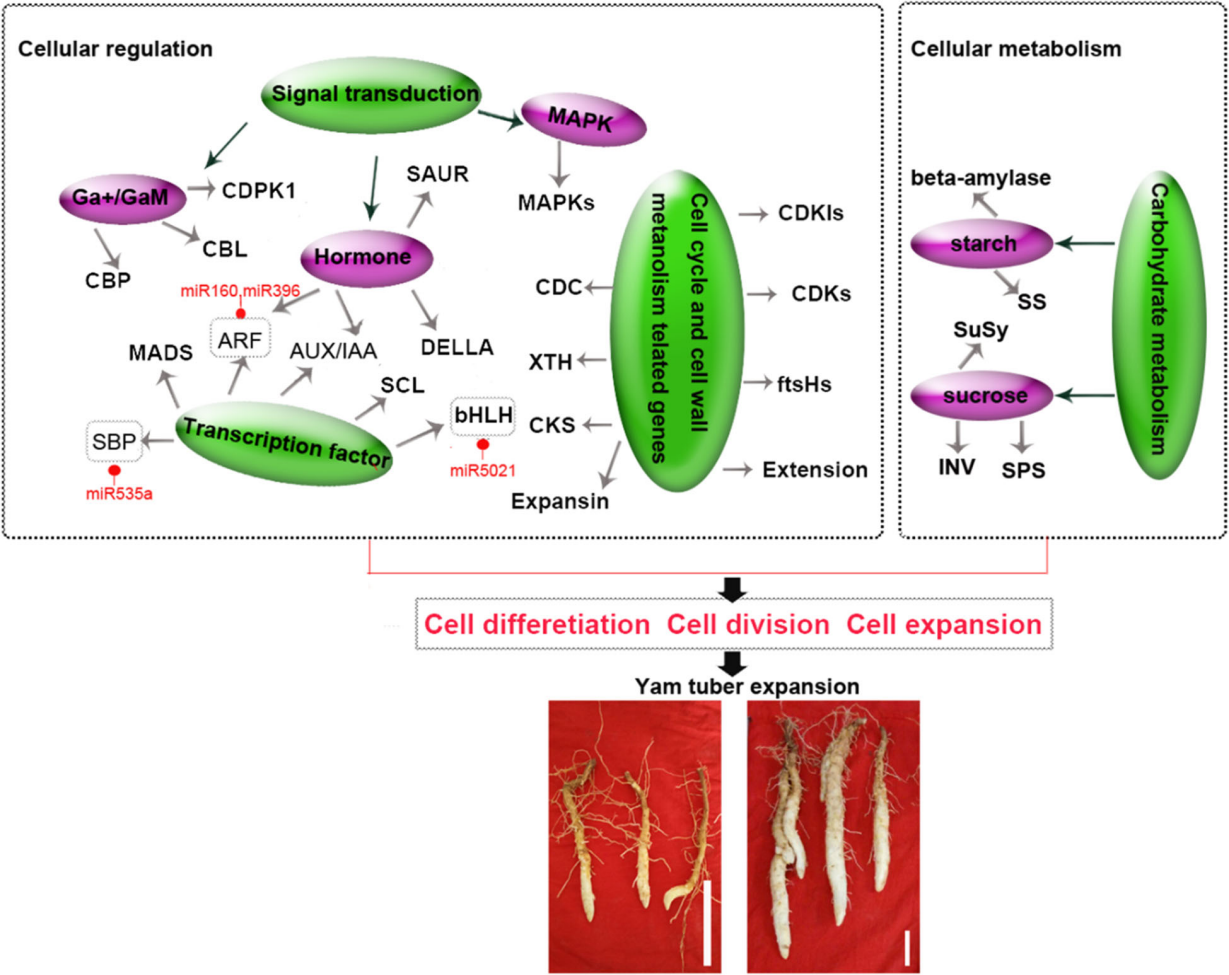
A proposed model of genetic and molecular interactions in the regulatory network during tuber expansion in yam. Arrows represents genes were involved in signal transduction pathways or carbohydrate metabolism, red circle represents target genes of miRNA

Tuber and root development and environmental responses involve gene regulatory networks [21, 24, 35, 37]. miRNA and target genes have been extensively studied in tuber development [21, 28], which significantly advanced our understanding of molecular regulatory networks underlying during yam tuber expansion. In this study, based on our RNA-Seq and small RNA analysis, previous transcriptomics analysis and results of other tuber crops, a putative model of regulatory network associated with yam tuber expansion was proposed (Fig. 8). During vegetative growth, environmental factors are the first signal to stimulate tuber growth, and cells development through some signal transduction pathways (hormone, calcium and, MAPK signaling) and metabolism processes (cell wall, starch and sucrose metabolism). Some specific genes promote cell differentiation division and expansion at tuber expansion. In detail, it was found that CBL, CBP, and GaM may affect cell division and proliferation during potato tubers expansion [40, 43, 46]. Moreover, MAPK signal pathway is known to play a central role in cell proliferation, differentiation and hormone signaling [50, 51]. XTH, expansin, extension, CKS, ftsHs, CDC5, CDC, CDKs, and CDKIs involved in cell division, cell expansion and cell cycles were differentially regulated during expansion stage. A finding similar to these differences in cell wall and cell cycle regulatory genes are involved in cell wall metabolism in *Arabidopsis*, *Rehmannia glutinosa*, radish [35, 36, 52]. DELLA controls cell expansion and cell division in hypocotyl, shoot, root, and floral induction [57]. DELLAs were significantly expressed in GA-mediated rhizome development process, suggesting that these DELLAs can regulate rhizomatic expansion [37]. In sweet potato, MADS were preferentially expressed in root at the initiation of the tuber, and in the vascular cambium region where the active cell proliferation was regulated by jasmonic acid, cytokinins, and stress response [53, 54]. Most Aux/IAA, ARFs and SAUR genes were enriched and expressed in the root cortex during splitting and expanding stage in radish, suggesting that these transcripts may be involved in cell expansion in the cambium [35]. SuSy, SPS, INV, SS, and beta-amylase, were highly correlated with tuber expansion, such as potato, radish and lotus [22, 35, 37]. In this study, some miRNA and target genes were observed during yam tuber expansion stage, bHLH (target by miR5021), SBP (target by miR535a), and ARFs (targeted by miRNA160 and miR396), were identified to be involved in cell expansion and cell differentiation in radish root and potato tuber [28, 35]. Taken together, the result suggested that these DEGs may play a vital role in the regulatory network of tuber expansion.

## Conclusion

A total of 14,238 genes expressed differentially during yam tuber expansion stage were identified first time using RNA-Seq technology. These results reveal the coordination of tuber involved in the processes of cellular events, metabolism, biosynthesis, and signal transduction pathways at transcriptional levels. It is worth noticing that the integrated analysis of miRNA-mRNA was identified to be differentially expressed in tuber expansion. The mRNA and miRNA datasets presented here identify a subset of candidate genes and miRNAs putatively associated with tuber expansion in yam, and therefore propose a hypothetical model of the genetic regulatory network associated with tuber expansion in yam, which can provide the basis for molecular regulatory mechanism to study the expansion development in *Dioscorea opposita* species.

## Methods

### Plant material

*Dioscorea opposita* cultivar Guihuai 16 was planted in April at the farm of Guangxi University in Nanning, Guangxi Province, China (22°53′06.7〞N 108°21′36.6〞E). Its healthy tubers germination, planting patterns and tuber growth stages were consistent as described by Gong [2] in 2016. All tuber growth stages were proved to have stable agronomic characteristics on three years of field observation. Tuber samples were collected at tuber initiation stage after field planting approximately forty days (initiation stage); additional samples were collected at sixty days (expansion stage). Anatomical characteristics of Guihuai 16 tuber at different developmental stages, including initiation and expansion stages were studied by Luo [67], and its histological characteristics consist of epidermis, parenchyma, and scattered vascular bundle. Five plants were selected randomly from every repetition each time. Then, the distal ends (5 mm long) of five fresh tubers from a repetition were washed with distilled water, cut down into pieces, and mixed as a biological repetition. All samples were immediately frozen in liquid nitrogen, and stored at − 80 °C until further use for the construction of various libraries. Two tuber developmental stages, including initiation and expansion stage, were used for the construction of various libraries. Three types of libraries were constructed, including one transcriptome de novo analysis libraries for mixed sample of initiation and expansion stage (named Total_1), six RNA-Seq analysis libraries (initiation stage named GH16_I, expansion stage named GH16_E), and small RNA analysis (initiation stage named C, and expansion stage named T in original data, in order to be consistent with the RNA-Seq data analyze, we renamed GH16_I, GH16_E for initiation and expansion stage, respectively). Each sample had three biological replicates with two technical replicates for each biological replicate.

### Construction and sequencing of transcriptome de novo, RNA-Seq and small RNA libraries

Information about the sample pool can be found on the subsections of plant materials above. Total RNA was isolated from distal ends of tuber samples using MiniBEST reagent (TaKaRa, Dalian, China), and RNA integrity was assessed by an Agilent 2100 BioAnalyzer. Construction of the transcriptome de novo and RNA-Seq libraries were performed at the Beijing Genome Institute (BGI; Shenzhen, China). Briefly, mRNA was purified from total RNA using poly-T oligo-attached magnetic beads, fragmented, and reverse transcribed into cDNA, and the full-length cDNA was used T4 DNA ligase (Invitrogen, USA), and products were enriched by PCR to generate the cDNA library. Finally, the cDNA library was examined using an Agilent 2100 Bioanalyzer before sequencing on BGISEQ-500 system platform at BGI-Shenzhen. Paired-end reads were generated with a length of 90 bp for each cDNA libraries.

For the small RNA libraries, RNA bands of around 18-30 nt (small RNAs) in length were isolated. Then small RNAs were ligated to a 5′-adaptor and a 3′-adaptor, and small RNAs were transcribed into cDNA. 14 rounds of PCR amplification were performed to enrich the cDNA fragments. After target fragments (100~120 bp) were purified, it was used for cluster generation and sequenced using BGISEQ-500 system platform at BGI-Shenzhen. BGISEQ-500 is a next-generation sequencing (NGS) technology platform developed by the Beijing Genomics Institute (BGI) in 2016 [68]. It is based on combinatorial Probe-Anchor Synthesis and improved DNA Nanoballs technology to generate short reads at a large scale. This platform has already been used for genome sequencing, RNA-Seq, and small RNA analysis.

Raw reads from mRNAs were subjected to remove the reads with adaptors or containing more than 5% unknown (‘N’) bases. Then, the low-quality reads (defined as reads having more than 20% of bases with quality≤15) were trimmed. The filtered clean reads were saved in FASTQ file, which has been submitted to Sequence Read Archive (SRA) of NCBI. Next, the Trinity software [69] was used to perform de novo assembly of the high-quality cleaned reads, and Tgicl software [70] was assembled to remove the redundant Trinity generated contigs. Final assembled datasets were performed to annotate genes function and expression.

Small RNA reads were also screened from raw sequencing reads by removing adaptors, poly-A sequences, and low-quality bases. Sequences shorter than 18 nt or longer than 32 nt were removed after trimming. Final filtered clean reads were saved in the FASTQ file, which was used for sequential analysis.

### Processing and mapping of mRNA-Seq and small RNA libraries sequencing data

After stringent quality filering, a mixed sample of initiation and expansion stage library and six RNA-Seq libraries clean reads were used to map the two reference genome sequences (*D. rotundata* and *D. alata,* bioproject accession numbers were PRJDB3383 and PRJEB10904, respectively). Among which, 40.91%~ 43.41% of reads were mapped to the *D. rotundata* genome, and 57.58%~ 61.16% to the *D. alata* genome (Additional file 9: Table S8). The two reference genomes were not plausible used for genes function and expression analysis. Hence functional databases NT, NR, GO, KOG, KEGG, SwissProt, and InterPro were used to annotate genes function, while Blastn, Blastx, Diamond, Blast2GO, and InterProScan5 were used to align genes. Six RNA-Seq reads were mapped to the transcriptome de novo analysis data (named Total_1) using bowtie2 results with default parameters after pre-processing mRNA-Seq data [71]. Gene expression levels were presented as FPKM (fragments per kilobase of exon per million fragments mapped) values [72], and the six small RNA clean reads were submitted to the sRNA databases by using Bowtie, Cmsearch, and Rfam software [73]. The clean reads aligned to the sRNA databases with more than 90% matched length and a maximum alignment score of 90 were used from further analysis.

### Identification of known and novel miRNAs, and predicting the targets of miRNAs

To identify the isoforms of known miRNA, cleaned sRNA reads were performed using the miRProf tool, and conserved miRNAs were identified using known plant miRNAs registered in miRBase [74]. To identify novel miRNAs and their precursors, unique reads were submitted to the RIPmiR [75]. Potential miRNA targets were predicted using the psRoot and Target Finder [76].

### Identification and functional annotation of differentially expressed genes and miRNAs

The expression levels of mRNA genes were measured as FPKM, and genes with expression levels > 5 FPKM were retained for statistical analysis. miRNA read counts were normalized to reads per million (TRM). Differentially expressed genes (DEGs) and miRNAs (DEMs) were identified by DEGseq [77]. Therefore, DEGs and DEMs were identified by stringent thresholds (|the FDR < 0.01, |log2 (fold change)| ≥ 1 and *P*-value < 0.005). All DEGs were subjected to gene ontology and KEGG pathway analysis. In addition, enrichment GO and KEGG pathway analyses were implemented using *p* ≤ 0.05 and q-value < 0.9 as the threshold. Enrichment analysis of DEGs was analyzed using GO and KEGG databases to obtain a detailed description of the DEGs. Each DEG in tuber was predicted by aligning the gene sequences against the Plant Transcriptional Factor Database. The DEGs were classified according to their TF families. A miRNA-target gene regulatory network was constructed using Cytoscape_v3. 2. 1 program.

### Validation of the DEGs and DEMs data using RT-qPCR

To identify the accuracy and reliability of mRNA and miRNA data, RT-qPCR was used to measure the expressions of DEGs and DEMs. Total RNA used for RNA-Seq and small RNA analysis previously was reversely transcribed into cDNA with PrimeScript™ RT reagent kit and Mir-X™ miRNA first-strand synthesis kit (Takara, Dalian, China), separately, according to the manufacture methods. RT-qPCR was performed using primers (Additional files 7 and 8: Tables S6 and S7) on real-time PCR detection system (BIO-RAD). Quantification expression for DEGs were detected by using the TB Green™ *Premix Ex Taq*™ II (Tli RNaseH Plus) (Takara, Dalian, China), 10 μl reaction solution containing 1x Green TB Premix Ex Taq II, 10 μM primer.

One-third dilution of the cDNA sample was used, and the reaction conditions were: 30s at 95 °C followed by 40 cycles of 30s at 95 °C and 30s at 60 °C. Quantification expression for DEMs were detected by the Mir-X miRNA qRT-PCR SYBR kit using 25 μl reaction solution, containing 2 × SYBR advantage premix, 50 × ROX dye, 10 μM miRNA-specific Primer, 2 μl cDNA samples were used and the reaction conditions were: 10s at 95 °C followed by 40 cycles of 5 s at 95 °C and 20s at 60 °C. All mRNAs and miRNA expressions had three biological replicates with three technical replicates for each of biological replicate, ACTIN was used as reference gene, the relative expression levels were collected by the comparative Ct protocol.

## Supplementary information

**Additional file 1: Figure S1.** Length distribution of small RNA sequences in small RNA libraries.

**Additional file 2: Table S1.** Differentially expressed genes (DEGs) in expansion stage compared with initiation stage.

**Additional file 3: Table S2.** The differentially expressed genes involved in signal transduction pathways in expansion stage (FPKM> 5).

**Additional file 4: Table S3.** The differentially expressed transcription factors involved in expansion stage.

**Additional file 5: Table S4.** Analysis of clean reads for small RNA sequencing in initiation and expansion stages.

**Additional file 6: Table S5.** Identification of miRNAs target genes in expansion stage.

**Additional file 7: Table S6.** The primers of 15 DEGs validated by RT-qPCR analysis.

**Additional file 8: Table S7.** The primers of 7 DEMs validated by RT-qPCR analysis.

**Additional file 9: Table S8.** Summary of alignment statistics of RNA-Seq libraries mapped to reference genome.

## Data Availability

The materials of this study were provided by the College of Agriculture at Guangxi University. Correspondence and requests for materials should be addressed to Longfei He (lfhe@gxu.edu.cn). The raw sequencing data have submitted to the NCBI SRA database (PRJNA533985). The assembled genes data also were submitted to GenBank TSA database (GHUN00000000).

## Abbreviations

B-ARR: myb family transcription factor
BKI1: BRI1 kinase inhibitor 1
BSK: BR-signaling kinase
CRE1: Farnesyl diphosphate synthase
CTR1: Serine/threonine-protein kinase CTR1
DEG: Differentially expressed gene
DEM: Differentially expressed microRNA
GA2ox: Gibberellin 2-beta-dioxygenase
GA3ox: Gibberellin 3-beta-dioxygenase
GO: Gene ontology
JAZ: Jasmonate ZIM domain-containing protein
KEGG: Kyoto encyclopedia of genes and genomes
miRNA: MicroRNA
MYC: Transcription factor MYC
NPR1: Regulatory protein NPR1
qRT-PCR: Real-time quantitative RT-PCR
RNA-Seq: RNA sequencing
SnRK2: Serine/threonine-protein kinase
PP2C: Protein phosphatase 2C
TF: Transcription factor
TGA: transcription factor TGA
