# Supplementary material for: Integrated mRNA and miRNA transcriptome analysis reveals a regulatory network for tuber expansion in Chinese yam (Dioscorea opposita)
Source: BMC Genomics. 2020 Feb 3;21:117. doi: 10.1186/s12864-020-6492-5 (PMC6998100; doi:10.1186/s12864-020-6492-5)
Supplement: Supplementary file 1 — Additional file 1: Figure S1. Length distribution of small RNA sequences in small RNA libraries. [file 12864_2020_6492_MOESM1_ESM.docx]

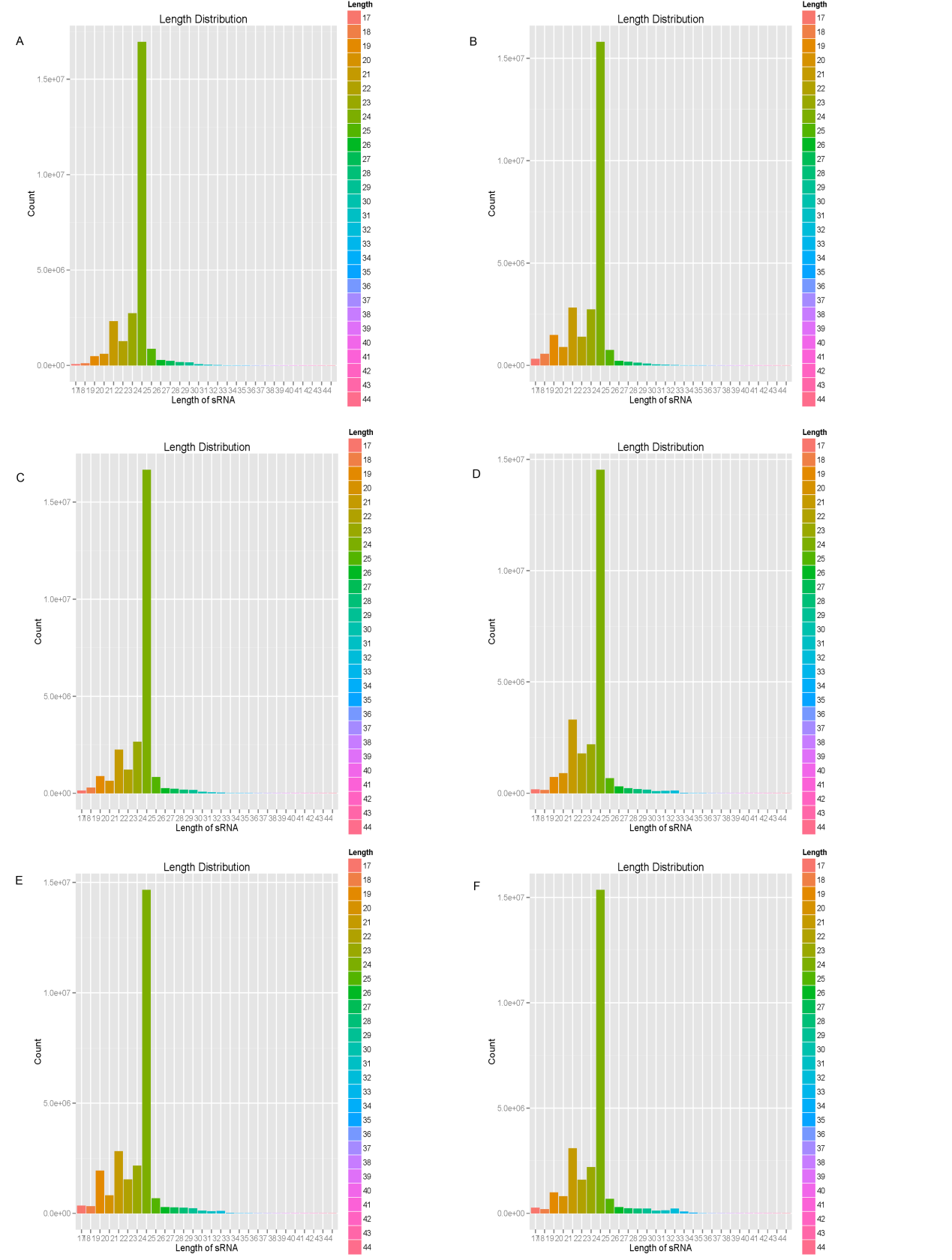


Fig S1: Length distribution of small RNA sequences in small RNA libraries.

A: GH16_I_r1 B: GH16_I_r2 C: GH16_I_r3 D: GH16_E_r1 E: GH16_E_r2

F: GH16_E_r3
